# Chronic voluntary wheel running exercise ameliorates metabolic dysfunction via PGC-1α expression independently of FNDC5/irisin pathway in high fat diet-induced obese mice

**DOI:** 10.1186/s12576-023-00864-6

**Published:** 2023-04-11

**Authors:** Chaeeun Cho, Minje Ji, Eunhee Cho, Seon Yi, Jae Geun Kim, Sewon Lee

**Affiliations:** 1 Department of Human Movement Science, Graduate School, Incheon National University, Incheon, Republic of Korea; 2 Department of Life Science, College of Life Sciences and Bioengineering, Incheon National University, Incheon, Republic of Korea; 3 Division of Sport Science, College of Arts & Physical Education, Incheon National University, Bldg# 16, Room# 423, (Songdo-Dong) 119 Academy-Ro, Yeonsu-Gu, Incheon, South Korea; 4 Sport Science Institute, College of Arts & Physical Education, Incheon National University, Incheon, Republic of Korea; 5 Health Promotion Center, College of Arts & Physical Education, Incheon National University, Incheon, Republic of Korea

**Keywords:** Exercise, Irisin, UCP1, PGC-1α, FNDC5

## Abstract

Exercise is an effective intervention to ameliorate metabolic diseases including obesity and insulin resistance, but the mechanisms involved in the metabolic amelioration have not yet been fully elucidated. This study aimed to determine whether AMPK–SIRT1–PGC-1α–FNDC5/Irisin-UCP1 expression is activated and whether metabolic dysfunction is ameliorated by chronic voluntary wheel running (VWR) in high-fat diet (HFD) induced obese mice. C57BL6J mice were randomly assigned into three groups at the age of 7 weeks for 10 weeks: normal chow diet (CON) group, HFD group, and HFD + VWR group. Chronic VWR ameliorates metabolic parameters and leads to increases in the expression of PGC-1α in the gastrocnemius muscle in HFD-induced obese mice. In contrast, the expression of AMPKα, SIRT1, and FNDC5, or circulating irisin levels did not lead to alteration. Improvement of metabolic health was partly mediated via PGC-1α expression by chronic VWR, but not FNDC5/Irisin pathway in HFD-induced obese mice.

**Supplementary Information:**

The online version contains supplementary material available at 10.1186/s12576-023-00864-6.

## Background

The increase in metabolic diseases including insulin resistance, metabolic syndrome, and diabetes mellitus worldwide is largely attributable to the global obesity epidemic [1–3]. Obesity is mainly caused by sedentary lifestyles and dietary habits, such as a high-fat diet (HFD) and a Western diet, in contemporary societies [4–7]. Various interventions have been proposed as methods to ameliorate obesity by increasing energy expenditure and promoting fat mobilization [8, 9].

Exercise is an effective intervention to ameliorate metabolic diseases including obesity, insulin resistance, and metabolic syndrome because exercise-induced muscle contraction stimulates the secretion of various bioactive hormones that contribute to beneficial effects on metabolic homeostasis [10–12]. The positive metabolic effects of exercise are influenced by AMP-activated protein kinase (AMPK) and Sirtuin 1 (SIRT1) expressed in skeletal muscle [13, 14]. AMPK and SIRT1 activation increase fatty acid oxidation and regulate whole-body energy metabolism by interacting with peroxisome proliferator-activated receptor-gamma coactivator 1-alpha (PGC-1α) [14–17]. PGC-1α expressed in skeletal muscle plays a pivotal role in maintaining metabolic function through glucose homeostasis, increased oxidative capacity, mitochondrial biogenesis, improved insulin sensitivity, suppressed muscle atrophy, and reduced systemic inflammation [18–20].

In skeletal muscle, PGC-1α regulates fibronectin type III domain-containing protein 5 (FNDC5) expression [21], which cleaved into irisin and released into the circulation [11]. It has been reported that the activation of the PGC-1α-FNDC5–Irisin axis enhances energy expenditure and increases the expression of thermogenic genes, such as uncoupling protein 1 (UCP1) [11, 22]. In this respect, exercise-induced activation of the AMPK–SIRT1–PGC-1α–FNDC5/Irisin–UCP1 axis can be an attractive therapeutic target for ameliorating metabolic diseases. Although previous studies have reported that AMPK and SIRT1 have a direct effect on PGC-1α activity and that PGC-1α regulates FNDC5/Irisin–UCP1, studies on whether chronic voluntary wheel running (VWR) exercise activates the AMPK–SIRT1–PGC-1α–FNDC5/Irisin–UCP1 series of signaling pathways are still lacking.

In addition, although previous studies have shown that exercise activates irisin-related signaling mechanisms, it is unclear whether 10 weeks of VWR exercise promotes this signaling mechanism and whether this activation pattern differs depending on the type of muscle fiber (Type I vs. Type I and II mixed). Therefore, this study aimed to confirm whether AMPK–SIRT1–PGC-1α–FNDC5/Irisin–UCP1 expression is stimulated and whether metabolic dysfunction is ameliorated by 10 weeks of VWR exercise in HFD-induced obese mice.

## Materials and methods

### Animal models

All experimental procedures were approved by the Animal Care Use Committee of the Incheon National University (INU-ANIM-2018-17). Six-week-old male wild-type C57BL6J mice were purchased from Jung Ang Lab Animal Inc. (Seoul, South Korea) and were housed in an animal facility conditioned with a temperature (20 ± 1 ℃), humidity (50–80%), and light-controlled on a 12-h light/dark cycle. All mice were acclimatized to the new animal facility for 1 week and were randomly assigned into three groups at the age of 7 weeks for 10 weeks: normal chow diet (control, CON, *n* = 14) group, high-fat diet (HFD, *n* = 14) group, and HFD with VWR (HFD + VWR, *n* = 14) group. The control group was placed on a standard chow diet that contained 3.5% fat (RodFeed, DBL, Inc.), while HFD and HFD + VWR groups were placed on the HFD that contained 20% carbohydrates, 20% protein, and 60% fat (D12492, Research Diets Inc, New Jersey, USA). All groups of mice were given access to a diet (normal chow or high-fat) and water ad libitum. Body mass was measured with an electronic scale (Mettler toledo, Switzerland) on a weekly basis.

### VWR exercise

VWR exercise monitoring was assessed in polycarbonate cages (20.5 cm wide × 36.5 cm long × 14 cm high) with free access to wheels (wheel diameter of 10.16 cm, interior diameter of 9.2 cm, wheel width of 5.1 cm, Columbus Instruments, Ohio, USA). CON and HFD groups were not treated with a running wheel. The total wheel running distance of the HFD + VWR group was recorded every 30 min for 24 h each day. The recorded revolutions were converted to km/day and recorded for 10 weeks.

### Measurement of blood parameters

Whole blood was obtained from tail vein after anesthesia with an intraperitoneal (IP) injection of 2.5% tribromoethanol (0.01 mL/g of body weight) to measure glucose and triglycerides levels in circulation. Blood glucose was measured using accu-check performa (Roche, South Korea) and triglycerides were measured using accutrend plus (Mannheim, Germany) by cutting the tail of the mouse before sacrifice and placing the blood on a blood test strip.

### Enzyme-linked immunosorbent assay (ELISA) analyses for measurement of insulin and irisin and HOMA-IR calculation

Serum samples were collected by extracting whole blood from the IP vena cava of anesthetized mouse and stored at room temperature for 30 min for coagulation. Then, the samples were centrifuged at 12,000 rpm for 10 min and stored at – 80 °C freezer until analysis. Commercially available ELISA kits were used according to the manufacturer's instructions to determine serum irisin (AdipoGen Life Sciences, San Diego, CA, USA, AG-45A-0046YEK-KI01) and insulin (ALPCO, Salem, NH, USA, 80-INSMS-E01) concentrations. All serum samples were measured in duplicate. Insulin resistance was determined by homeostatic model assessment for insulin resistance (HOMA-IR) and HOMA-IR formula is: HOMA-IR = (glucose [mmol/L] × (insulin [mU/L])/22.5 [23].

### Tissue collection

Gastrocnemius and soleus muscles were isolated from both legs, and brown adipose tissue (BAT) was isolated from the interscapular region of mice. To isolate BAT, the dorsal interscapular region of the mice was incised, the butterfly shaped interscapular adipose tissue was separated, and then the white adipose tissue was removed. To isolate gastrocnemius and soleus muscles, the skin on both legs of the mice was peeled off and the skeletal muscles of the lower extremity were exposed. Then, the tendon was incised and the gastrocnemius was isolated. After the separation of the gastrocnemius muscle, the soleus muscle was exposed and the soleus muscle isolated by incising the tendon.

### Western blot analyses

The collected skeletal muscles and fat tissues were homogenized with CelLytic MT lysis buffer (Sigma-Aldrich) mixed with protease inhibitors cocktail (Sigma-Aldrich) at 1:100 ratio. Protein amounts from all samples were assessed using the BCA protein assay kit (Thermo Scientific) followed by protein concentration normalization prior to all western blot experiments. The same amount of protein was separated with SDS–PAGE in 12% or 7.5% polyacrylamide gel and then transferred to PVDF (Bio-Rad, CA, USA). Membranes were blocked for 1 h at room temperature in blocking solution (5% skim milk) followed by overnight incubation (4 °C) in primary antibody diluted in blocking solution (5% Bovine Serum Albumin). Membranes were probed using the following antibodies: total FNDC5 (Abcam, catalog# ab-174833, 1:1000), PGC‐1α (Abcam, catalog# ab-54481, 1:1000), UCP1 (Abcam, catalog# ab-10983, 1:1000), AMPKα (Cell signaling, catalog#2562, 1:1000), SIRT1 (Cell signaling, catalog# 9475, 1:500), and β‐actin (Santa Cruz Biotechnology, catalog# sc-47778, 1:1000). Following TBST washes, FNDC5, PGC-1α, UCP1, AMPKα, SIRT1, and β‐actin were incubated for 1 h at room temperature with secondary antibodies (Abcam, 1:2000). All bands were visualized by enhanced chemiluminescence. FNDC5, PGC-1α, UCP1, AMPKα, SIRT1, and β‐actin bands were detected and quantified using Bio‐Rad ChemiDoc Touch Imaging System (Bio-Rad Laboratories, Hercules, CA, USA) with Image Software Lab.

### Statistical analysis

All statistical analyses were evaluated using GraphPad Prism (version 9.0, GraphPad Software, USA). All variables were presented as a mean ± SEM and normality of distribution for variables was assessed using the Shapiro–Wilk test. The comparison of every week for the body weight was performed by two-way mixed model (group x time) ANOVA with repeated measurements. The remaining variables were performed by one-way ANOVA, and in the case of variables not following the normality, Kruskal–Wallis test was performed. Intergroup differences were performed with Bonferroni post-hoc comparisons. The correlations were performed via Pearson’s correlation analysis, and in the case of variables not following the normality, Spearman’s correlation analysis was performed. The significance level was *P* < 0.05.

## Results

### Effects of HFD and VWR exercise on weight, blood glucose, triglycerides, insulin, and HOMA-IR

**Fig. 1:**
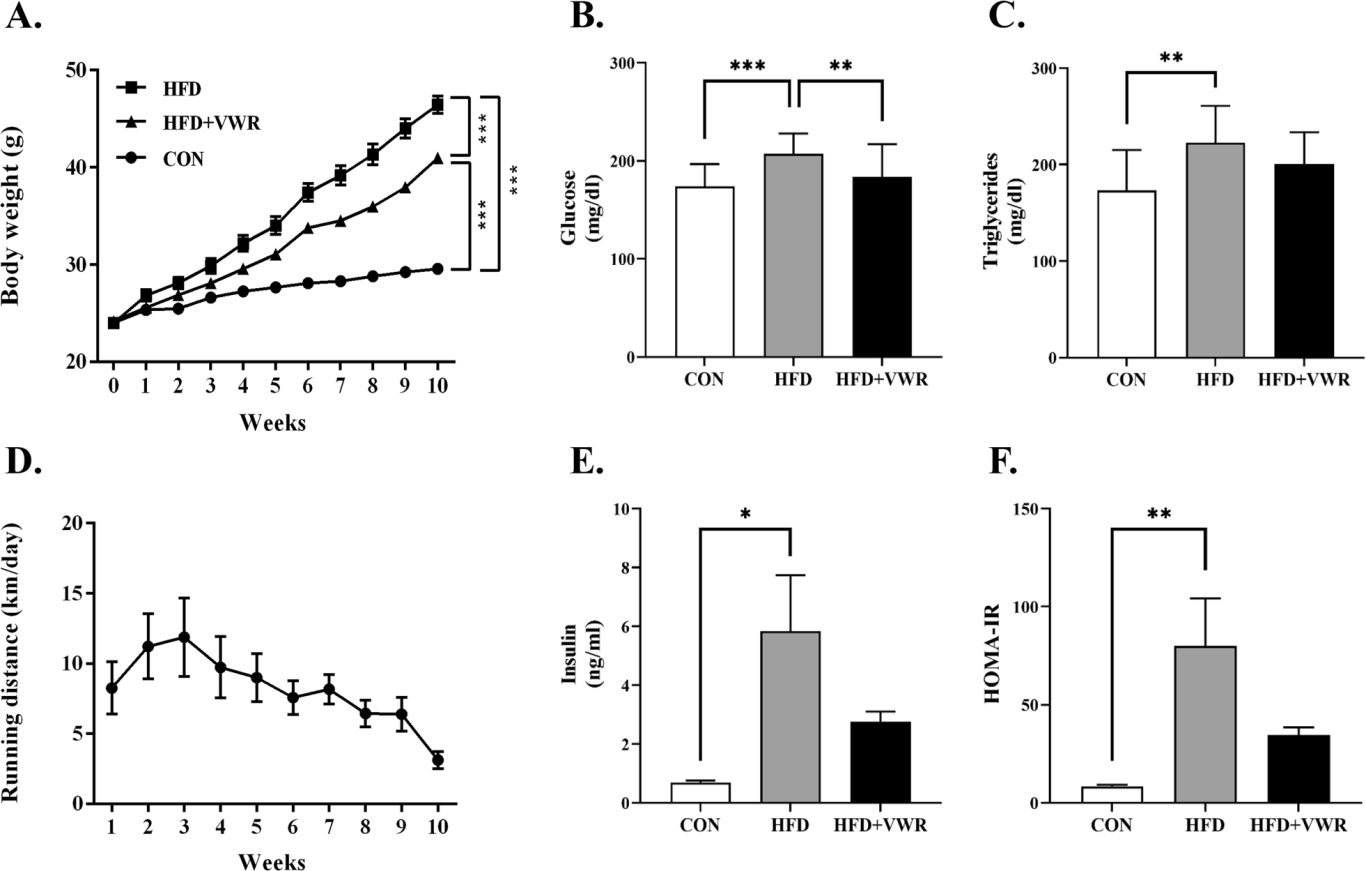
Effects of HFD and VWR exercise on weight **A**, blood glucose **B**, triglycerides **C**, running distance **D**, insulin **E**, and HOMA-IR **F**. Values are shown as mean ± SEM. *P < .05, **P < .01, ***P < .001

After 10 weeks of diet and VWR exercise intervention, the HFD and HFD + VWR groups had significantly increased weight compared to the CON group (both *P* < 0.0001), but the HFD + VWR group had significantly lower weight compared to the HFD group (*P* < 0.0001, Fig. 1A). The HFD group showed significantly increased blood glucose (Fig. 1B), triglycerides (Fig. 1C), insulin (Fig. 1E), and HOMA-IR (Fig. 1F) levels compared to the CON group. Furthermore, the HFD group had significantly higher blood glucose levels than that of the HFD + VWR group (Fig. 1B). However, when compared to the CON group, HFD + VWR group did not show an increase in blood glucose, triglycerides, insulin, or HOMA-IR indicating that VWR exercise prevented weight gain and improved metabolic parameters in HFD-induced obese mice. The average daily voluntary wheel exercise distance in HFD + VWR group was 8.25 ± 0.8 km (Fig. 1D).

### Effects of chronic VWR exercise on AMPKα, SIRT1, PGC-1α, and FNDC5 in the gastrocnemius and soleus muscles

**Fig. 2:**
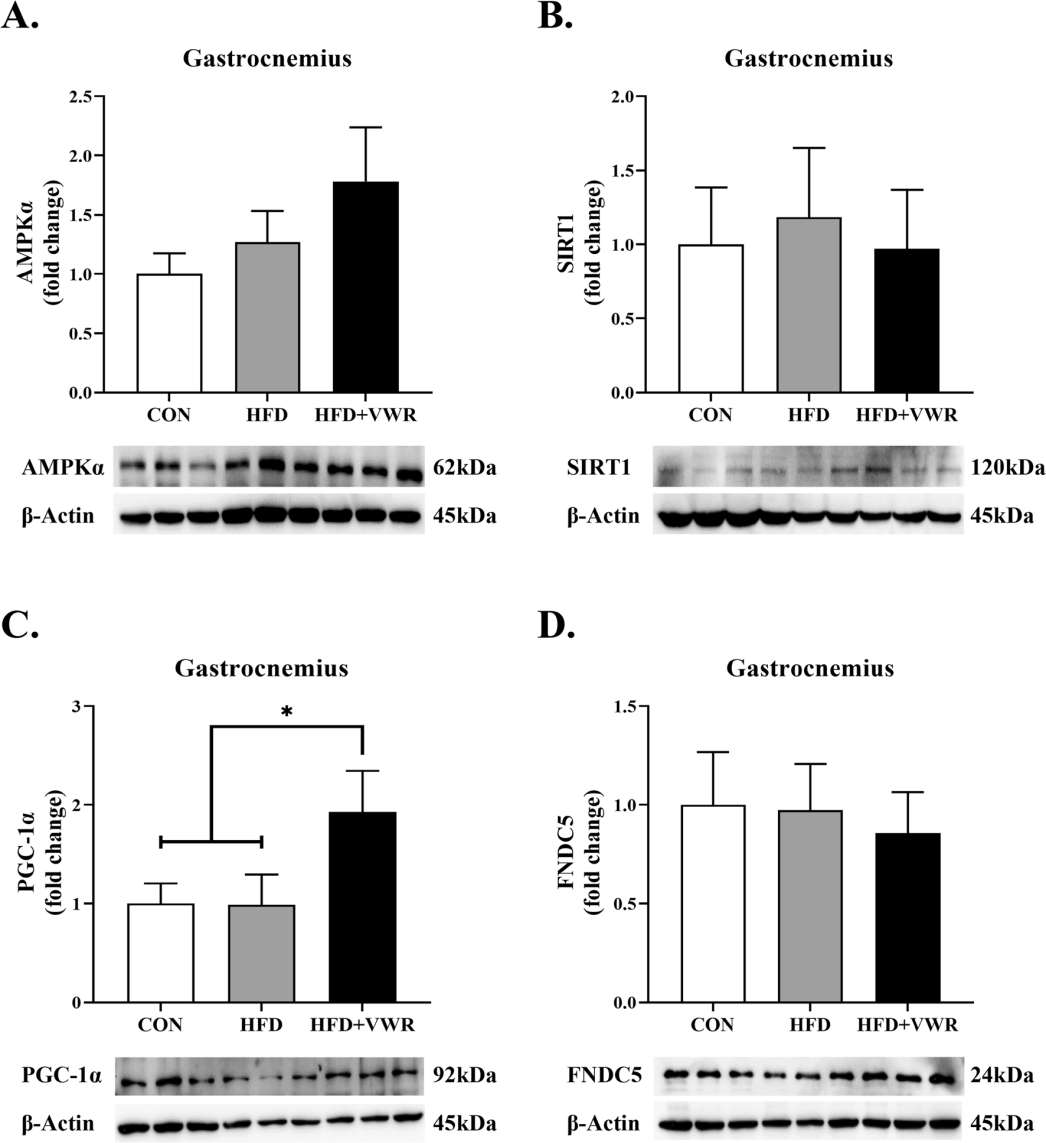
Effects of HFD and VWR exercise on AMPKα **A**, SIRT1 **B**, PGC-1α **C**, and FNDC5 **D** in the gastrocnemius muscles. Values are shown as mean ± SEM. *P < .05

**Fig. 3:**
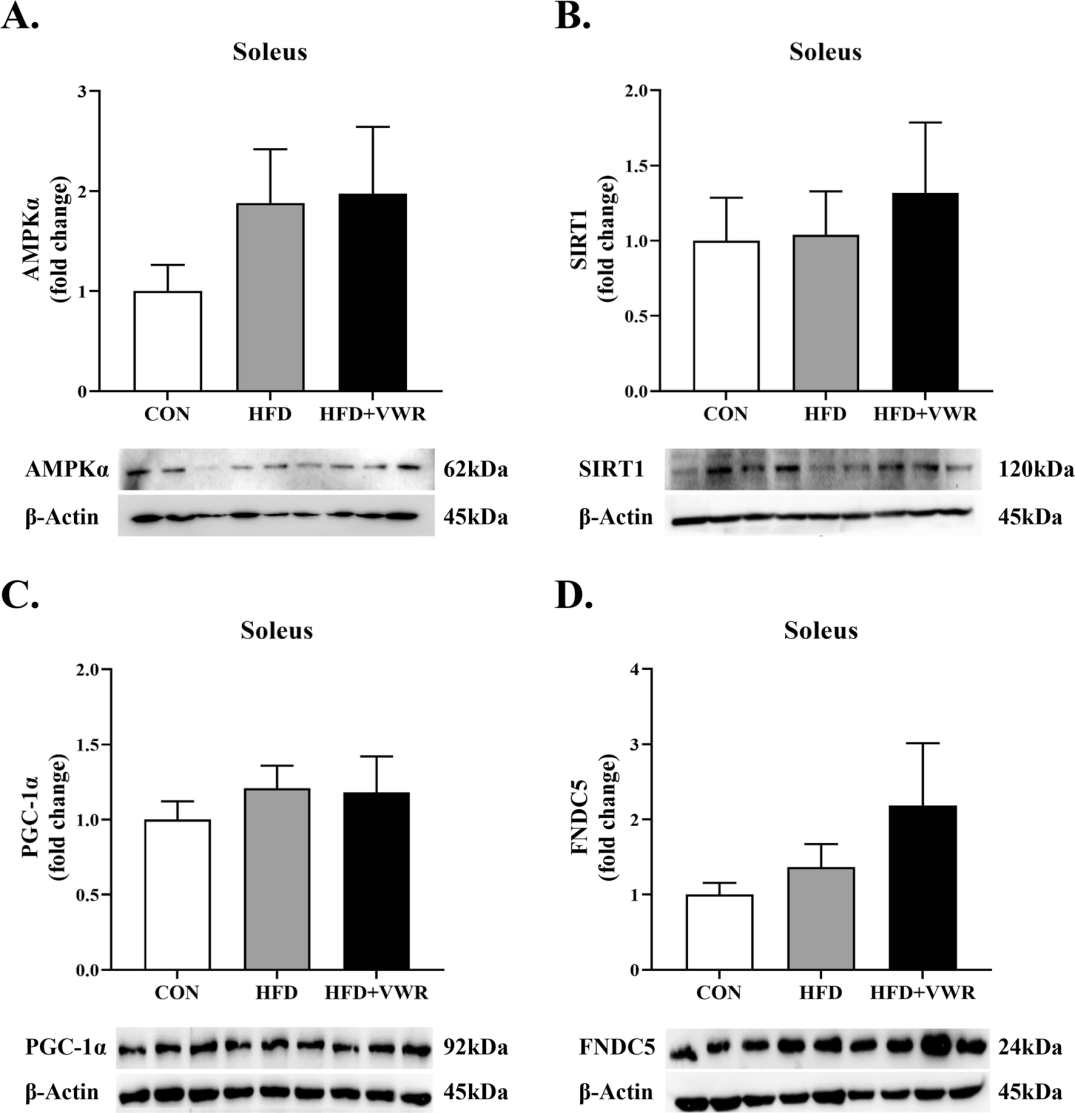
Effects of HFD and VWR exercise on AMPKα **A**, SIRT1 **B**, PGC-1α **C**, and FNDC5 **D** in the soleus muscles. Values are shown as mean ± SEM

To determine whether chronic WVR exercise activates AMPKα, SIRT1, PGC-1α, and FNDC5 in the skeletal muscles, we measured protein expression in the gastrocnemius and soleus muscles which were type I and II mixed muscle fiber and type I muscle fiber, respectively. The expression levels of AMPKα (Fig. 2A) and SIRT1 (Fig. 2B) in the gastrocnemius muscle were comparable among the groups, with a tendency toward an increase in AMPKα expression in the HFD + VWR group, but the difference was not statistically significant. The expression of PGC-1α in the gastrocnemius muscle of HFD + VWR group was significantly higher than that in the CON and HFD groups (Fig. 2C). However, the expression of FNDC5 in the gastrocnemius muscle was comparable among the groups (Fig. 2D). Expression levels of AMPKα, SIRT1, PGC-1α, and FNDC5 were also examined in the soleus muscle. The expression levels of AMPKα (Fig. 3A), SIRT1 (Fig. 3B), PGC-1α (Fig. 3C), and FNDC5 (Fig. 3D) in the soleus muscles were comparable among the groups.

### Effects of chronic VWR exercise on irisin in serum

**Fig. 4:**
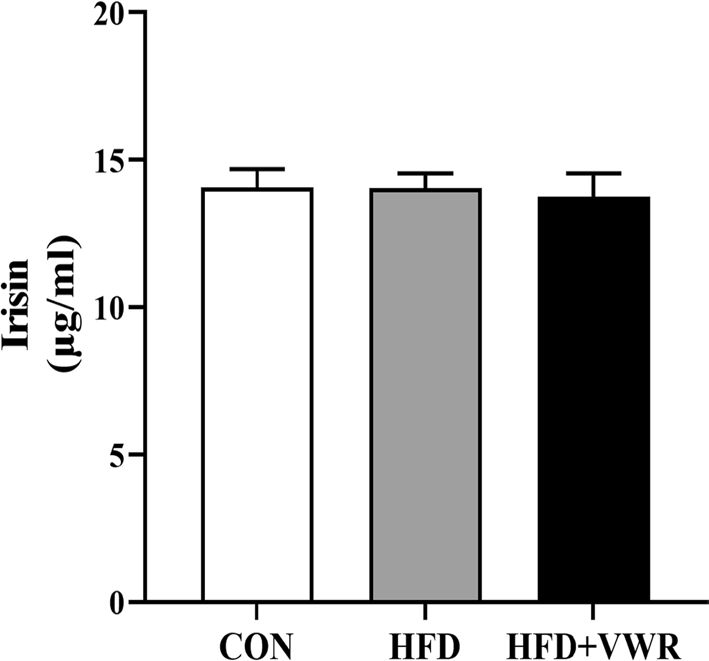
Effects of chronic HFD and VWR exercise on irisin in serum. Values are shown as mean ± SEM

To test whether chronic VWR exercise increases circulating irisin levels, we measured irisin concentration using ELISA. The concentrations of irisin were comparable among the groups. Chronic VWR exercise did not lead to an increase in circulating irisin levels (Fig. 4).

### Effects of chronic VWR exercise on UCP1 in BAT

**Fig. 5:**
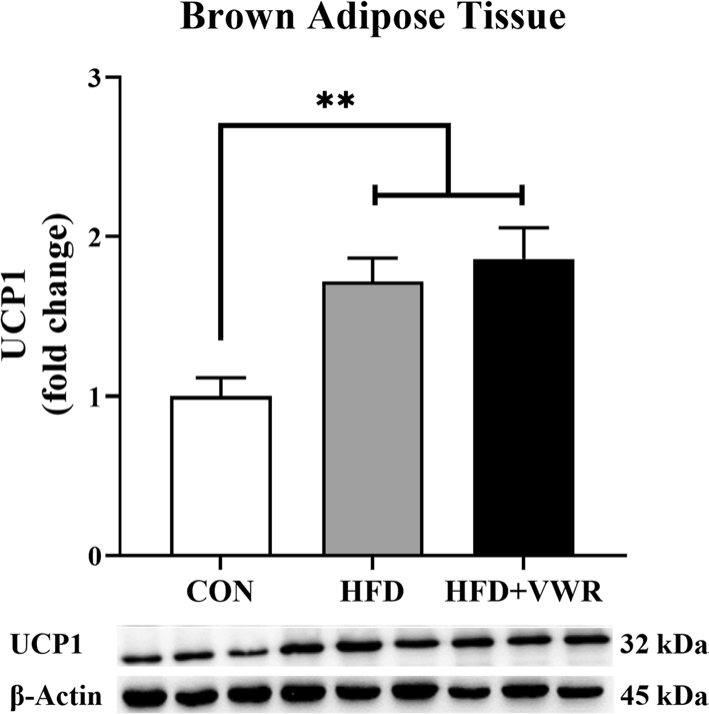
Effects of chronic HFD and VWR exercise on UCP1 in brown adipose tissue (BAT). Values are shown as mean ± SEM. **P < .01

To test whether chronic VWR exercise increases UCP1 expression, we examined its expression in BAT. UCP1 expression was significantly higher in HFD and HFD + VWR groups compared to the CON group (Fig. 5). However, no significant differences in UCP1 expression were found between the HFD and HFD + VWR groups.

### Correlation analysis between expressed proteins in the gastrocnemius and soleus muscles

**Fig. 6:**
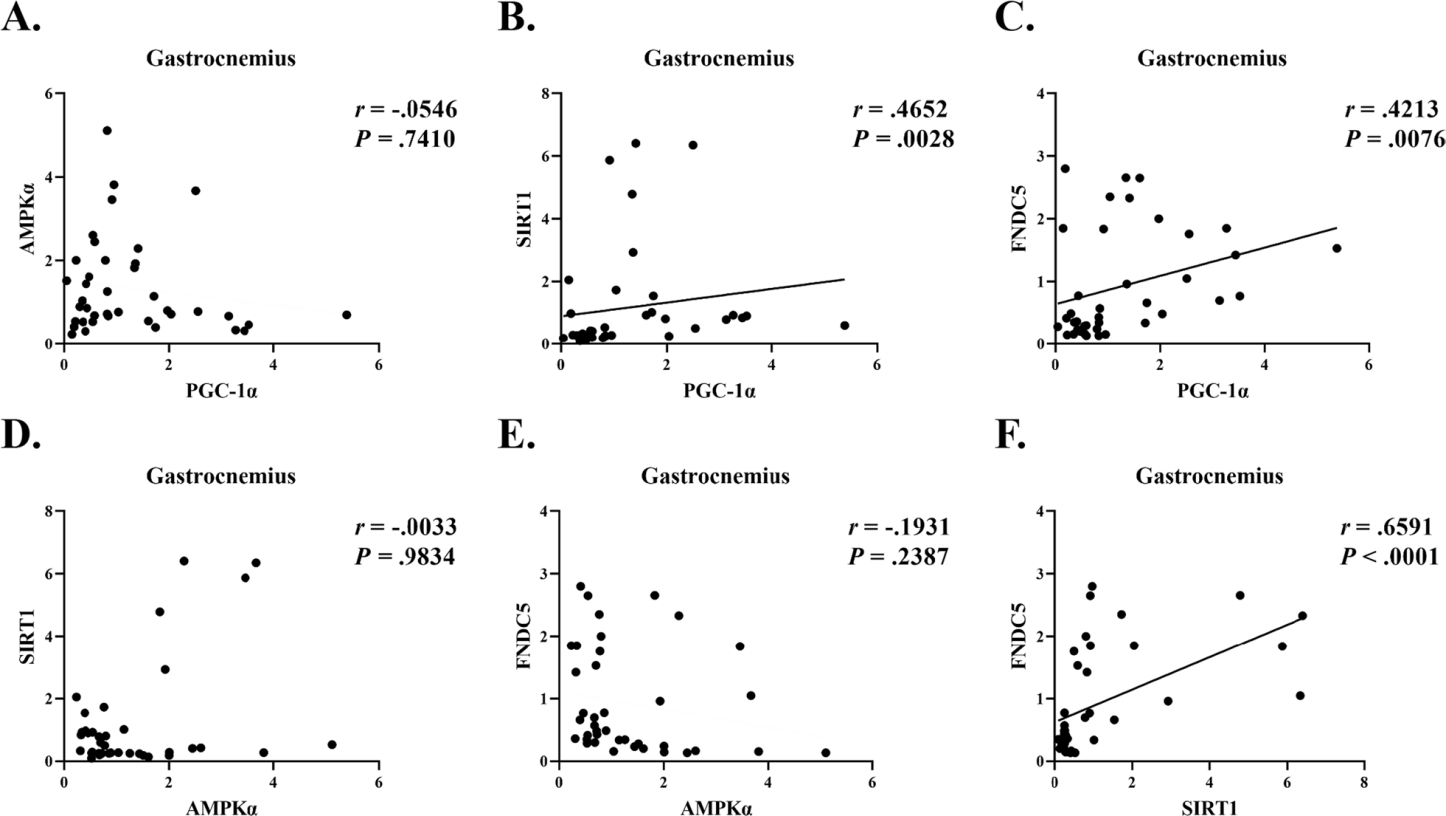
Correlation analysis between expressed proteins in the gastrocnemius muscles

**Fig. 7:**
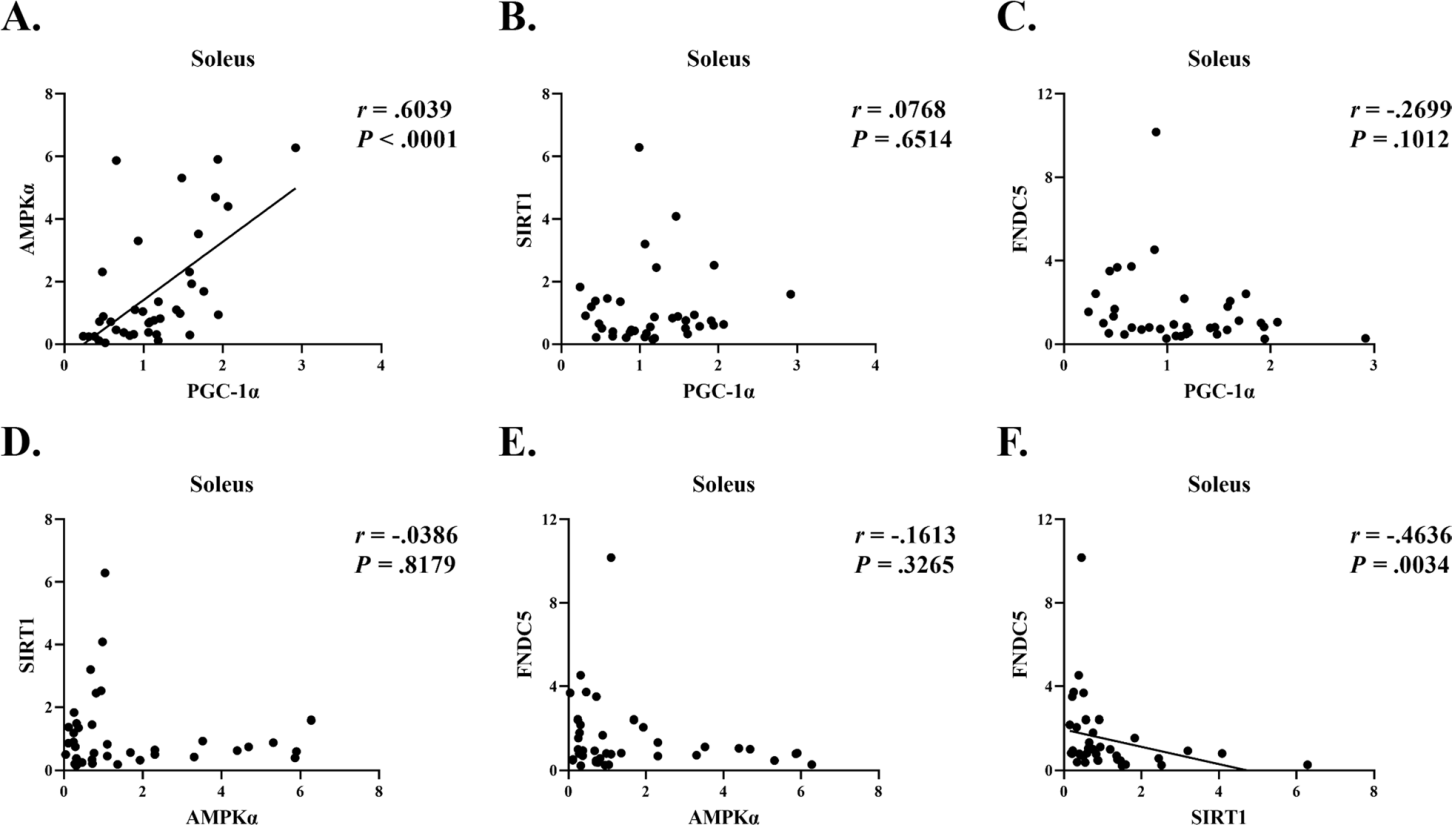
Correlation analysis between expressed proteins in the soleus muscles

A previous study reported that PGC-1α regulates FNDC5 activation in response to chronic exercise and that PGC-1α shows a positive correlation with FNDC5 in skeletal muscle [24]. However, the activation pattern of PGC1α and FNDC5 differed according to muscle fiber type after exercise [25, 26]. Therefore, in this study, a correlation analysis was conducted to investigate the protein expression patterns according to muscle fiber types. The results of the correlations in the gastrocnemius muscles showed a significant positive correlation between SIRT1, PGC-1α, and FNDC5 (Fig. 6F, C, respectively). Furthermore, a significant positive correlation was observed between the SIRT1 and PGC-1α expression (Fig. 6B). However, no correlation was found between SIRT1, PGC-1α, FNDC5, and AMPKα (Fig. 6A, E, D, respectively). A correlation analysis was also performed between proteins expressed in the soleus muscle. A positive correlation was observed between AMPKα and PGC-1α expression (Fig. 7A). However, a negative correlation was found between SIRT1 and FNDC5 expression (Fig. 7F). Furthermore, the remaining variables showed no correlation (Fig. 7B, C, D and E, respectively).

## Discussion

This study aimed to determine whether AMPK–SIRT1-PGC-1α–FNDC5/Irisin–UCP1 expression, which is an irisin-related signaling pathway, was activated and whether metabolic dysfunction was ameliorated by 10 weeks of chronic VWR exercise in HFD-induced obese mice. In this study, chronic VWR exercise prevented weight gain and improved metabolic risk factors, such as blood glucose, triglycerides, insulin, and HOMA-IR. Although no alterations in FNDC5/Irisin were observed in response to chronic VWR exercise, a significantly increased expression of PGC-1α protein was found only in the gastrocnemius muscle which has both type I and II muscle fibers. Furthermore, increased PGC-1α protein expression was associated with increased SIRT1 and FNDC5 in the gastrocnemius muscle, but not in the soleus muscle which has many type I muscle fibers. These results suggest that protein expression patterns may differ depending on muscle fiber type. The protein expression of UCP1 in BAT was significantly increased in the HFD and HFD + VWR groups compared to the CON group.

A sedentary lifestyle and type 2 diabetes mellitus are associated with decreased expression of PGC-1α in the skeletal muscle [20, 27]. Conversely, increased expression of PGC-1α in skeletal muscle is known to ameliorate metabolic factors, such as insulin sensitivity and insulin resistance [28, 29]. The increased expression of PGC-1α induced by exercise in the skeletal muscles may differ depending on the muscle fiber type, exercise type, and exercise duration. One previous study found that when mice were treated with VWR exercise for 1, 2, 4, 6, and 8 weeks, the expression of PGC-1α was increased only in the plantaris muscle which has type II muscle fiber, but not in the soleus muscle which has type I muscle fibers [25]. In addition, no significant alteration of PGC-1α in the plantaris muscle was evident in the 1 and 2 week VWR exercise groups, whereas a significant increase was observed in the long-term (4, 6, and 8 weeks) voluntary wheel exercise groups [25]. In this study, PGC-1α expression in the gastrocnemius muscle, but not in the soleus muscle, may have increased by chronic VWR exercise, because the gastrocnemius muscle has a type I and II mixed muscle fiber type, whereas the soleus muscle has a type I muscle fiber. Therefore, these results may suggest that increased PGC-1α protein levels in the skeletal muscle are associated with long-term exercise and type II muscle fiber rather than short-term exercise and type I muscle fiber.

Previous studies indicated that exercise-induced AMPK activation led to up-regulation of PGC-1α expression in the skeletal muscle [15, 30, 31]. However, in this study, the protein expression of AMPKα did not increase in the gastrocnemius and soleus muscles. According to these findings, it is possible that other molecules, such as the p38 mitogen-activated protein kinase (MAPK) signaling pathway, but not AMPK and SIRT1, expressed PGC-1α through VWR exercise. Aerobic exercise in rodents and humans increases the phosphorylation of p38 MAPK in the skeletal muscle [32–35]. In addition, VWR exercise-induced activation of p38 MAPK has been shown to be involved in the regulation of PGC-1α in mouse skeletal muscle [36]. In this regard, the expression of PGC-1α may be activated by the p38-MAPK signaling pathway induced by chronic VWR exercise in HFD-induced obese mice. However, further studies are needed to investigate the mechanisms involved in these signaling pathways.

BAT is characterized by the presence of small fat-filled droplets and large numbers of mitochondria [37]. This is known to increase energy consumption via thermogenesis [38]. BAT deficiency induces obesity, whereas increased BAT levels regulate glucose homeostasis and improve insulin sensitivity [39, 40]. Therefore, increased thermogenesis in BAT may be a potential therapeutic target for ameliorating obesity and metabolic risk factors. The thermogenic capacity of BAT is demonstrated via activated UCP1, which dissipates the chemical energy of fatty acids as heat [38, 41–43]. In addition, exercise stimulates BAT and induces UCP1 expression [44]. In this study, we found that UCP1 levels were significantly increased in the HFD and HFD + VWR groups compared to the CON group, but no difference was observed between the HFD and HFD + VWR groups. Increased UCP1 levels in BAT may be a response to a HFD. Several studies have reported that a HFD increases ketogenesis and induces obesity, insulin resistance, and systemic hyperlipidemia [45, 46]. In addition, a HFD leads to increased blood ketones and a darker color by feeding ketone esters, decreasing lipid droplet size, and increasing the number of mitochondria in BAT [47]. Giraud et al. demonstrated that the HFD group had significantly increased UCP1 mRNA levels in BAT compared to the standard diet group and the high-carbohydrate diet group [48]. Therefore, it is suggested that the significantly increased UCP1 levels in BAT may be an effect of increased ketone bodies induced by HFD.

Irisin, an exercise-induced myokine, is known to provide several metabolic benefits, such as ameliorating glucose homeostasis by reducing insulin resistance [49]. However, the effects of exercise on irisin are still under discussion, because different results have been reported depending on the intensity and duration of exercise. In this study, chronic VWR exercise did not alter FNDC5 protein or circulating irisin levels. The PGC-1α protein in the gastrocnemius muscle was increased without alteration in the levels of FNDC5/Irisin. In contrast, the results showed a positive correlation between SIRT1, PGC-1α, and FNDC5 in the gastrocnemius muscle. However, VWR exercise may not be sufficient to stimulate FNDC5/Irisin. Previous studies found that VWR exercise for 4 weeks did not increase FNDC5 levels in mouse gastrocnemius muscle [50], and chronic moderate exercise (VO_2_ max 70%) did not increase levels of circulating irisin in humans [24]. Interestingly, irisin levels in the skeletal muscle were not altered by moderate-intensity aerobic exercise (HRpeak 65%), but significantly increased after high-intensity interval training (six bouts of 1 min at HRpeak 85–95%) in humans [51]. In addition, a recent study reported that high-intensity interval training elicits a higher peak circulating irisin response than moderate-intensity continuous aerobic exercise [52]. Given these previous findings, exercise intensity may be related to the FNDC5/Irisin signaling pathways, and VWR exercise may have been insufficient to activate these signaling pathways. Furthermore, irisin may be associated with exercise duration. Previous studies showed increased levels of FNDC5 expression by acute swimming and treadmill exercise in the mouse skeletal muscle and increased levels of irisin by 3 weeks of voluntary wheel exercise in mouse plasma [11, 26, 53]. In a network meta-analysis study, acute exercise showed the greatest potential as the best intervention to improve the levels of irisin in humans [54]. In addition, irisin has been shown to be a molecule with a short half-life and high degradation rate [24, 55, 56]. For these reasons, irisin response after exercise may be conflicted by exercise intensity and duration.

This study has some limitations. First, we analyzed only the total AMPKα protein as a signaling pathway. There was no alteration in the total AMPKα protein, but phosphorylated AMPKα may be activated via exercise. Therefore, future studies are needed to investigate the ratio of phosphorylated AMPKα to total AMPKα protein. Second, the normal chow diet with a VWR (CON + VWR) group was not assigned. Since there is no CON + VWR group in this study, it may be insufficient to logically explain protein changes caused by exercise without the effect of a HFD. Third, food intake was not measured in this study. According to previous studies, high-intensity exercise reduced appetite and food intake [57, 58], whereas VWR increased food intake and decreased plasma leptin levels and the weight of adipose tissue [59]. These previous findings indicate that a decrease in food intake may not have been seen in the HFD + VWR group in this study. However, not measuring food intake is a limitation of this study and it may be necessary to record food intake in follow-up studies. This is because changes in body weight and metabolic parameters may be dependent on food intake. Finally, the protein expression in the gastrocnemius muscle was analyzed without separating the white and red regions. Although we performed mRNA analysis by separating the red and white regions of the gastrocnemius muscle as a preliminary study after this study, the mRNA levels of PGC-1α and FNDC5 in the red and white regions of the gastrocnemius muscle were comparable between the groups (Additional file 1). However, in a previous study, the protein expression of PGC-1α was increased by 6 h of prolonged running exercise in the red region of the rat gastrocnemius muscle, but not in the white region of the rat gastrocnemius muscle [60]. Therefore, in future studies, it will be necessary to investigate the signaling pathways in the white and red regions of the gastrocnemius muscle in more detail.

## Conclusions

In summary, the chronic VWR exercise did not alter the protein expression of AMPKα, SIRT1, and FNDC5 in both soleus and gastrocnemius muscles, or circulating irisin when compared to the control group. In contrast, chronic VWR exercise leads to increase in the expression of PGC-1α in the gastrocnemius muscle. Furthermore, chronic VWR exercise ameliorates weight gain and metabolic parameters, such as blood glucose, triglycerides, and insulin resistance in HFD-induced obese mice. This study showed that chronic VWR exercise ameliorates metabolic health via PGC-1α expression independently of FNDC5/Irisin pathway in HFD-induced obese mice.

### Supplementary Information

**Additional file 1. Figure S1. **Effects of HFD and VWR exercise on mRNA expression of PGC-1α (A), FNDC5 (B) in white region of gastrocnemius muscle and PGC-1 α (C), FNDC5 (D) in red region of gastrocnemius muscle.

## Data Availability

The data that support the findings of this study are available from the corresponding author upon reasonable request.

## Abbreviations

AMPK: AMP-activated protein kinase
BAT: Brown adipose tissue
CON: Normal chow diet
ELISA: Enzyme-linked immunosorbent assay
FNDC5: Fibronectin type III domain-containing protein 5
HFD: High-fat diet
HOMA-IR: Homeostatic model assessment for insulin resistance
IP: Intraperitoneal
MAPK: Mitogen-activated protein kinase
PGC-1α: Peroxisome proliferator-activated receptor-gamma coactivator 1-alpha
SIRT1: Sirtuin 1
UCP1: Uncoupling protein 1
VWR: Voluntary wheel running

## References

[1] Kopelman PG (2000). Obesity as a medical problem. Nature.

[2] Swinburn BA, Sacks G, Hall KD, McPherson K, Finegood DT, Moodie ML, Gortmaker SL (2011). The global obesity pandemic: shaped by global drivers and local environments. Lancet.

[3] Sundström-Poromaa I, Thu WPP, Kramer MS, Logan S, Cauley JA, Yong EL (2020). Risk factors for insulin resistance in midlife Singaporean women. Maturitas.

[4] Silveira EA, Mendonça CR, Delpino FM, Elias Souza GV, de Souza P, Rosa L, de Oliveira C, Noll M (2022). Sedentary behavior, physical inactivity, abdominal obesity and obesity in adults and older adults: a systematic review and meta-analysis. Clin Nutr ESPEN.

[5] Sousa LGO, Marshall AG, Norman JE, Fuqua JD, Lira VA, Rutledge JC, Bodine SC (2021). The effects of diet composition and chronic obesity on muscle growth and function. J Appl Physiol.

[6] Schrauwen P, Westerterp KR (2000). The role of high-fat diets and physical activity in the regulation of body weight. Br J Nutr.

[7] Naja F, Hwalla N, Itani L, Karam S, Sibai AM, Nasreddine L (2015). A Western dietary pattern is associated with overweight and obesity in a national sample of Lebanese adolescents (13–19 years): a cross-sectional study. Br J Nutr.

[8] Bae JH, Lee H (2021). The effect of diet, exercise, and lifestyle intervention on childhood obesity: a network meta-analysis. Clin Nutr.

[9] Seo YG, Lim H, Kim Y, Ju YS, Lee HJ, Jang HB, Park SI, Park KH (2019). The effect of a multidisciplinary lifestyle intervention on obesity status, body composition, physical fitness, and cardiometabolic risk markers in children and adolescents with obesity. Nutrients.

[10] Korkmaz A, Venojärvi M, Wasenius N, Manderoos S, Deruisseau KC, Gidlund EK, Heinonen OJ, Lindholm H, Aunola S, Eriksson JG, Atalay M (2019). Plasma irisin is increased following 12 weeks of Nordic walking and associates with glucose homoeostasis in overweight/obese men with impaired glucose regulation. Eur J Sport Sci.

[11] Boström P, Wu J, Jedrychowski MP, Korde A, Ye L, Lo JC, Rasbach KA, Boström EA, Choi JH, Long JZ, Kajimura S, Zingaretti MC, Vind BF, Tu H, Cinti S, Højlund K, Gygi SP, Spiegelman BM (2012). A PGC1-α-dependent myokine that drives brown-fat-like development of white fat and thermogenesis. Nature.

[12] Huh JY (2018). The role of exercise-induced myokines in regulating metabolism. Arch Pharm Res.

[13] Liao ZY, Chen JL, Xiao MH, Sun Y, Zhao YX, Pu D, Lv AK, Wang ML, Zhou J, Zhu SY, Zhao KX, Xiao Q (2017). The effect of exercise, resveratrol or their combination on Sarcopenia in aged rats via regulation of AMPK/Sirt1 pathway. Exp Gerontol.

[14] Rodgers JT, Lerin C, Haas W, Gygi SP, Spiegelman BM, Puigserver P (2005). Nutrient control of glucose homeostasis through a complex of PGC-1alpha and SIRT1. Nature.

[15] Jäger S, Handschin C, St-Pierre J, Spiegelman BM (2007). AMP-activated protein kinase (AMPK) action in skeletal muscle via direct phosphorylation of PGC-1alpha. Proc Natl Acad Sci U S A.

[16] Cantó C, Auwerx J (2009). PGC-1alpha, SIRT1 and AMPK, an energy sensing network that controls energy expenditure. Curr Opin Lipidol.

[17] Lee WJ, Kim M, Park HS, Kim HS, Jeon MJ, Oh KS, Koh EH, Won JC, Kim MS, Oh GT, Yoon M, Lee KU, Park JY (2006). AMPK activation increases fatty acid oxidation in skeletal muscle by activating PPARalpha and PGC-1. Biochem Biophys Res Commun.

[18] Sandri M, Lin J, Handschin C, Yang W, Arany ZP, Lecker SH, Goldberg AL, Spiegelman BM (2006). PGC-1alpha protects skeletal muscle from atrophy by suppressing FoxO3 action and atrophy-specific gene transcription. Proc Natl Acad Sci U S A.

[19] Benton CR, Nickerson JG, Lally J, Han XX, Holloway GP, Glatz JF, Luiken JJ, Graham TE, Heikkila JJ, Bonen A (2008). Modest PGC-1alpha overexpression in muscle in vivo is sufficient to increase insulin sensitivity and palmitate oxidation in subsarcolemmal, not intermyofibrillar, mitochondria. J Biol Chem.

[20] Handschin C, Spiegelman BM (2008). The role of exercise and PGC1alpha in inflammation and chronic disease. Nature.

[21] Shimba Y, Togawa H, Senoo N, Ikeda M, Miyoshi N, Morita A, Miura S (2019). Skeletal muscle-specific PGC-1α overexpression suppresses atherosclerosis in apolipoprotein E-knockout mice. Sci Rep.

[22] Zhang Y, Li R, Meng Y, Li S, Donelan W, Zhao Y, Qi L, Zhang M, Wang X, Cui T, Yang LJ, Tang D (2014). Irisin stimulates browning of white adipocytes through mitogen-activated protein kinase p38 MAP kinase and ERK MAP kinase signaling. Diabetes.

[23] Zhang H, Park Y, Zhang C (2010). Coronary and aortic endothelial function affected by feedback between adiponectin and tumor necrosis factor α in type 2 diabetic mice. Arterioscler Thromb Vasc Biol.

[24] Norheim F, Langleite TM, Hjorth M, Holen T, Kielland A, Stadheim HK, Gulseth HL, Birkeland KI, Jensen J, Drevon CA (2014). The effects of acute and chronic exercise on PGC-1α, irisin and browning of subcutaneous adipose tissue in humans. Febs j.

[25] Ikeda S, Kawamoto H, Kasaoka K, Hitomi Y, Kizaki T, Sankai Y, Ohno H, Haga S, Takemasa T (2006). Muscle type-specific response of PGC-1 alpha and oxidative enzymes during voluntary wheel running in mouse skeletal muscle. Acta Physiol (Oxf).

[26] Namgoong H, Lee J-s, Kim J-G, Lee S (2018). Acute effects of aerobic treadmill exercise intensity on expression of irisin and FNDC5 in male mouse. Exerc Sci.

[27] Wu H, Deng X, Shi Y, Su Y, Wei J, Duan H (2016). PGC-1α, glucose metabolism and type 2 diabetes mellitus. J Endocrinol.

[28] Benton CR, Holloway GP, Han XX, Yoshida Y, Snook LA, Lally J, Glatz JF, Luiken JJ, Chabowski A, Bonen A (2010). Increased levels of peroxisome proliferator-activated receptor gamma, coactivator 1 alpha (PGC-1alpha) improve lipid utilisation, insulin signalling and glucose transport in skeletal muscle of lean and insulin-resistant obese Zucker rats. Diabetologia.

[29] Lira VA, Benton CR, Yan Z, Bonen A (2010). PGC-1alpha regulation by exercise training and its influences on muscle function and insulin sensitivity. Am J Physiol Endocrinol Metab.

[30] Terada S, Goto M, Kato M, Kawanaka K, Shimokawa T, Tabata I (2002). Effects of low-intensity prolonged exercise on PGC-1 mRNA expression in rat epitrochlearis muscle. Biochem Biophys Res Commun.

[31] Zeng Z, Liang J, Wu L, Zhang H, Lv J, Chen N (2020). Exercise-induced autophagy suppresses sarcopenia through Akt/mTOR and Akt/FoxO3a signal pathways and AMPK-mediated mitochondrial quality control. Front Physiol.

[32] Goodyear LJ, Chang PY, Sherwood DJ, Dufresne SD, Moller DE (1996). Effects of exercise and insulin on mitogen-activated protein kinase signaling pathways in rat skeletal muscle. Am J Physiol.

[33] Kramer HF, Goodyear LJ (2007). Exercise, MAPK, and NF-kappaB signaling in skeletal muscle. J Appl Physiol.

[34] Nader GA, Esser KA (2001). Intracellular signaling specificity in skeletal muscle in response to different modes of exercise. J Appl Physiol.

[35] Widegren U, Jiang XJ, Krook A, Chibalin AV, Björnholm M, Tally M, Roth RA, Henriksson J, Wallberg-henriksson H, Zierath JR (1998). Divergent effects of exercise on metabolic and mitogenic signaling pathways in human skeletal muscle. Faseb j.

[36] Akimoto T, Pohnert SC, Li P, Zhang M, Gumbs C, Rosenberg PB, Williams RS, Yan Z (2005). Exercise stimulates Pgc-1alpha transcription in skeletal muscle through activation of the p38 MAPK pathway. J Biol Chem.

[37] Sanchez-Delgado G, Martinez-Tellez B, Olza J, Aguilera CM, Gil Á, Ruiz JR (2015). Role of exercise in the activation of brown adipose tissue. Ann Nutr Metab.

[38] Cannon B, Nedergaard J (2004). Brown adipose tissue: function and physiological significance. Physiol Rev.

[39] Hamann A, Flier JS, Lowell BB (1996). Decreased brown fat markedly enhances susceptibility to diet-induced obesity, diabetes, and hyperlipidemia. Endocrinology.

[40] Stanford KI, Middelbeek RJ, Townsend KL, An D, Nygaard EB, Hitchcox KM, Markan KR, Nakano K, Hirshman MF, Tseng YH, Goodyear LJ (2013). Brown adipose tissue regulates glucose homeostasis and insulin sensitivity. J Clin Invest.

[41] Oelkrug R, Polymeropoulos ET, Jastroch M (2015). Brown adipose tissue: physiological function and evolutionary significance. J Comp Physiol B.

[42] Richard D, Picard F (2011). Brown fat biology and thermogenesis. Front Biosci.

[43] Klingenspor M, Herzig S, Pfeifer A (2012). Brown fat develops a brite future. Obes Facts.

[44] Ringholm S, Grunnet Knudsen J, Leick L, Lundgaard A, Munk Nielsen M, Pilegaard H (2013). PGC-1α is required for exercise- and exercise training-induced UCP1 up-regulation in mouse white adipose tissue. PLoS ONE.

[45] Sikder K, Shukla SK, Patel N, Singh H, Rafiq K (2018). High fat diet upregulates fatty acid oxidation and ketogenesis via intervention of PPAR-γ. Cell Physiol Biochem.

[46] Sunny NE, Satapati S, Fu X, He T, Mehdibeigi R, Spring-Robinson C, Duarte J, Potthoff MJ, Browning JD, Burgess SC (2010). Progressive adaptation of hepatic ketogenesis in mice fed a high-fat diet. Am J Physiol Endocrinol Metab.

[47] Veech RL (2013). Ketone esters increase brown fat in mice and overcome insulin resistance in other tissues in the rat. Ann N Y Acad Sci.

[48] Giraudo SQ, Kotz CM, Grace MK, Levine AS, Billington CJ (1994). Rat hypothalamic NPY mRNA and brown fat uncoupling protein mRNA after high-carbohydrate or high-fat diets. Am J Physiol.

[49] Perakakis N, Triantafyllou GA, Fernández-Real JM, Huh JY, Park KH, Seufert J, Mantzoros CS (2017). Physiology and role of irisin in glucose homeostasis. Nat Rev Endocrinol.

[50] Guilford BL, Parson JC, Grote CW, Vick SN, Ryals JM, Wright DE (2017). Increased FNDC5 is associated with insulin resistance in high fat-fed mice. Physiol Rep.

[51] Archundia-Herrera C, Macias-Cervantes M, Ruiz-Muñoz B, Vargas-Ortiz K, Kornhauser C, Perez-Vazquez V (2017). Muscle irisin response to aerobic vs HIIT in overweight female adolescents. Diabetol Metab Syndr.

[52] Colpitts BH, Rioux BV, Eadie AL, Brunt KR, Sénéchal M (2022). Irisin response to acute moderate intensity exercise and high intensity interval training in youth of different obesity statuses: a randomized crossover trial. Physiol Rep.

[53] Cho E, Jeong DY, Kim JG, Lee S (2021). The acute effects of swimming exercise on PGC-1α-FNDC5/irisin-UCP1 expression in male C57BL/6J mice. Metabolites.

[54] Kazeminasab F, Sadeghi E, Afshari-Safavi A (2022). Comparative impact of various exercises on circulating irisin in healthy subjects: a systematic review and network meta-analysis. Oxid Med Cell Longev.

[55] Nygaard H, Slettaløkken G, Vegge G, Hollan I, Whist JE, Strand T, Rønnestad BR, Ellefsen S (2015). Irisin in blood increases transiently after single sessions of intense endurance exercise and heavy strength training. PLoS ONE.

[56] Hecksteden A, Wegmann M, Steffen A, Kraushaar J, Morsch A, Ruppenthal S, Kaestner L, Meyer T (2013). Irisin and exercise training in humans—results from a randomized controlled training trial. BMC Med.

[57] Deighton K, Karra E, Batterham RL, Stensel DJ (2013). Appetite, energy intake, and PYY3-36 responses to energy-matched continuous exercise and submaximal high-intensity exercise. Appl Physiol Nutr Metab.

[58] Thivel D, Isacco L, Montaurier C, Boirie Y, Duché P, Morio B (2012). The 24-h energy intake of obese adolescents is spontaneously reduced after intensive exercise: a randomized controlled trial in calorimetric chambers. PLoS ONE.

[59] Yasumoto Y, Nakao R, Oishi K (2015). Free access to a running-wheel advances the phase of behavioral and physiological circadian rhythms and peripheral molecular clocks in mice. PLoS ONE.

[60] Terada S, Tabata I (2004). Effects of acute bouts of running and swimming exercise on PGC-1alpha protein expression in rat epitrochlearis and soleus muscle. Am J Physiol Endocrinol Metab.

