# Supplementary material for: Chronic voluntary wheel running exercise ameliorates metabolic dysfunction via PGC-1α expression independently of FNDC5/irisin pathway in high fat diet-induced obese mice
Source: J Physiol Sci. 2023 Apr 11;73:6. doi: 10.1186/s12576-023-00864-6 (PMC10717694; doi:10.1186/s12576-023-00864-6)
Supplement: Supplementary file 1 — Additional file 1. Figure S1. Effects of HFD and VWR exercise on mRNA expression of PGC-1α (A), FNDC5 (B) in white region of gastrocnemius muscle and PGC-1 α (C), FNDC5 (D) in red region of gastrocnemius muscle. [file 12576_2023_864_MOESM1_ESM.pptx]

## Slide 1
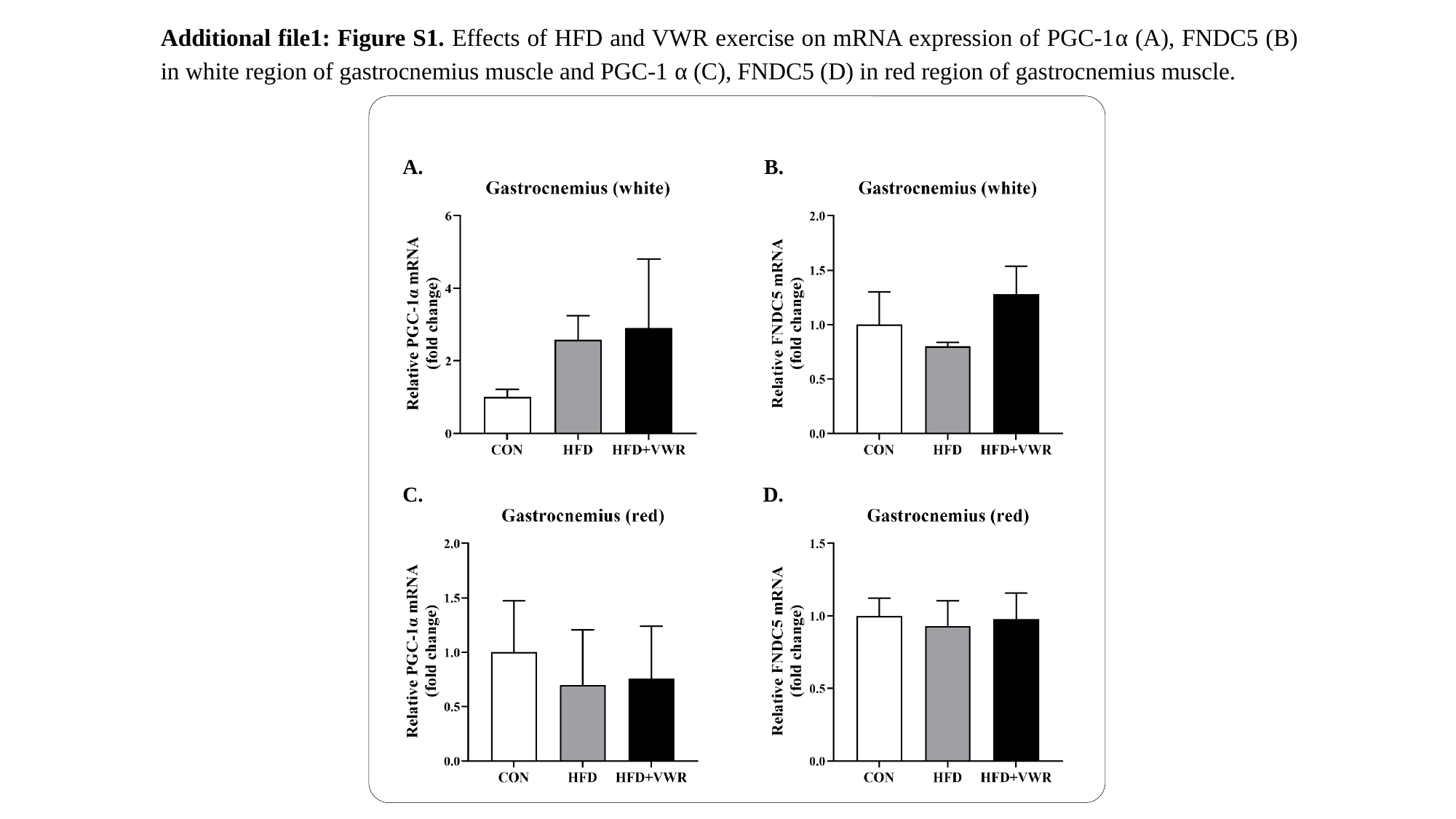

Additional file1: Figure S1. Effects of HFD and VWR exercise on mRNA expression of PGC-1α (A), FNDC5 (B) in white region of gastrocnemius muscle and PGC-1 α (C), FNDC5 (D) in red region of gastrocnemius muscle.
A.
B.
C.
D.
